# Supplementary figures and images for: Clinical characteristics and factors associated with long COVID among post-acute COVID-19 clinic patients in Zambia, August 2020 to January 2023: A cross-sectional and longitudinal study design
Source: PLoS One. 2024 Jul 2;19(7):e0306131. doi: 10.1371/journal.pone.0306131 (PMC11219000; doi:10.1371/journal.pone.0306131)

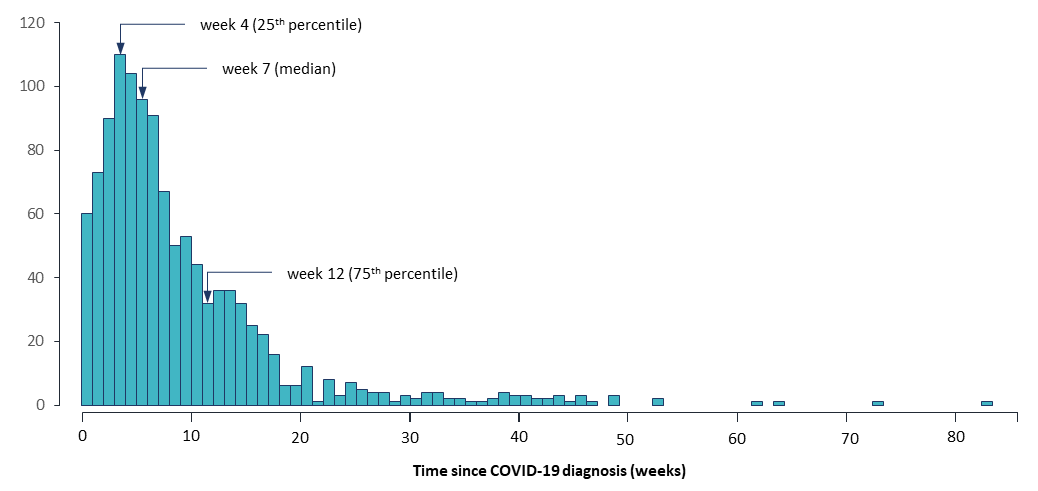

Supplement: S1 Fig — (TIF) [file pone.0306131.s001.tif]
