# Supplementary material for: Clinical characteristics and factors associated with long COVID among post-acute COVID-19 clinic patients in Zambia, August 2020 to January 2023: A cross-sectional and longitudinal study design
Source: PLoS One. 2024 Jul 2;19(7):e0306131. doi: 10.1371/journal.pone.0306131 (PMC11219000; doi:10.1371/journal.pone.0306131)
Supplement: Attachment — Submitted filename: Response to reviewers.docx [file pone.0306131.s006.docx]

Below are the responses to reviewers and how each comment was addressed by the authors:

| **#** | **Comment** | **Details on how comment has been addressed** | **Manuscript line number** |
| --- | --- | --- | --- |
| 1. | Please ensure that your manuscript meets PLOS ONE's style requirements, including those for file naming. The PLOS ONE style templates | Manuscript has been updated per PLOS guidelines. This included abstract headings to level 1, sentence case for headings, figure and table citations, author affiliation information, among others | Entire document |
| 2. | Please provide an amended statement that declares *all* the funding or sources of support (whether external or internal to your organization) received during this study, | Provided updated funding statement with **all** funders and included in the cover letter | Cover letter |
| 3. | Please remove any funding-related text from the manuscript and let us know how you would like to update your Funding Statement | Funding statement received from manuscript and updated stamen provided in cover letter | Cover letter |
| 4. | Your ethics statement should only appear in the Methods section of your manuscript. If your ethics statement is written in any section besides the Methods, please delete it from any other section. | Ethics statement moved to methods section | 179-188 |
| 5. | Please include a separate caption for each figure in your manuscript. | Separate captions included | 199, 205, & 268 |
| 6. | Please include captions for your Supporting Information files at the end of your manuscript, and update any in-text citations to match accordingly. Please see our Supporting Information guidelines for more information: http://journals.plos.org/plosone/s/supporting-information. | Updated supporting information provided and intext citation updated. | 256, 209, 262, 286-287, 373-388; |
| 7. | Please include your tables as part of your main manuscript and remove the individual files. Please note that supplementary tables (should remain/ be uploaded) as separate "supporting information" files | Tables and figures include in main manuscript and removed individual files. Supporting information retained as separate files | Entire document |
| 8. | Background: The background section needs to highlight the study's relevance and clearly identify the existing knowledge gap that the research aims to address. | Background section updated to highlight the study's relevance and clearly identify the existing knowledge. | 49-96 |
| 9. | The methods section should be more comprehensive, providing crucial details such as the study population, duration, key variables, and a thorough description of the analyses performed. | Study design, participants, design, variables, study size, and statistical analysis have now been included in the manuscript | 98-178 |
| 10. | Conclusion: Ensure that the conclusion strictly aligns with the study's objective, summarizing the key findings and their implications in a way that directly responds to the research aim. | Conclusion updated to align with objectives | 360-370 |
| 11. | The introduction is brief and lacks exploration of the topic, failing to address the knowledge gap. It is crucial to clarify the study's relevance and contribution. Are there limited related studies, and if so, why is this important? What advances does the current study offer compared to existing research? Elaborating on these aspects will provide a stronger foundation for the study's significance. | Addressed as described in part (8) above | 49-96 |
| 12. | The authors further state: “While dozens of seroprevalence studies in Africa document the extent of spread of SARS-CoV-2...”. However, they only cite two studies, review. Once again, I emphasize that the lack of studies should not be the justification for carrying out the research, as even if there were few, it may have been sufficient. To review. | Statement reviewed as per comment | 91-93 |
| 13. | It is imperative to enhance the clarity and completeness of the methods section by adhering to the observational study checklist (STROBE). This checklist should encompass the following details: study design, setting, participants, variables, data sources/measurement, bias, study size, quantitative variables, and statistical methods. The STROBE checklist can be accessed at <https://www.strobe-statement.org/checklists/>. | Addressed as described in part (8) above | 49-96 |
| 14. | Furthermore, the location of the study is unclear. Was data collected from all the mentioned hospitals? Please review and rephrase for clarity. | Study site information provided | 99-101 |
| 15. | Regarding [supplementary] Table 2, it is unclear if the authors conducted tests other than calculating the 95% confidence interval. Was the Chi-square test with Bonferroni correction performed? Please provide clarification on this matter. | For the longitudinal analysis which involved 5 PAC-19 clinic data points, we corrected for pairwise comparisons using Bonferroni correction. Notes include in the table updated to highlight the change | S1 Table |
| 16. | The statement, "...with data entered in the electronic database were included in the analyses," is more appropriate for the methods section rather than the results. | Statement moved to method’s section | 190 |
| 17. | Regarding the authors' statement, "Of these inpatients, 686 (74.6%) were classified as having had severe illness," it is crucial to define what constitutes severe illness for better understanding. | Severe illness rewritten as “severe COVID” and definition provide accordingly. | 133-134 |
| 18. | If available, consider including data on the number of vaccine doses received by patients, as this information could be relevant to the study. | Data on vaccine doses included | 229-236 |
| 19. | It is not clear whether sex, vaccination status, and referral to a specialized service were included in the final model. The authors should specify whether these factors were part of the final model in the results section. | Sex included in the model | Table 2, Table 3, S3 Table and S4 Table |
| 20 | The first paragraph in the discussion should serve as a concise summary of the main results, aligning with the study's objective, which will be elaborated on subsequently. Ensure that the discussion follows the sequence of the results. | Paragraph updated and written to be concise | 294-300 |
| 21. | For the fourth paragraph, the mention of bias should be removed. The final paragraph of the discussion is the appropriate space to discuss the study's limitations, including any biases. | Bias removed from paragraph 4 and moved to limitation paragraphs | 347-361 |
| 22. | Regarding the vaccination aspect, expand the discussion by exploring what other studies reveal about the vaccine. Compare and contrast findings with existing literature. Additionally, delve into the question of how the number of vaccine doses might be associated, emphasizing the importance of including and discussing these results. Refer to relevant literature on this topic. | Discussion on vaccination has been expanded also limitation on vaccination dates restricted classification of vaccination status as full, partial, indeterminate, or unvaccinated. | 320-327 |
| 23. | LIMITS: The authors omit a crucial point regarding the local representativeness of the data. It's imperative to acknowledge that the findings may not be extrapolated to other locations or even the entire country, given that the study is based solely on a sample from one institution. Additionally, there is ambiguity regarding the specific hospitals from which the data were collected, requiring clarification for transparency. Please, clarify what are the strengths of this work. | Manuscript updated with limitation on representativeness of the study | 347-354 |
| 24 | The conclusion requires careful review to ensure strict alignment with the study's objectives. Therefore, the main findings of the study must initially be added. Afterwards, other questions can be raised | Conclusion updated | 363-372 |
