# Supplementary material for: Clinical characteristics and factors associated with long COVID among post-acute COVID-19 clinic patients in Zambia, August 2020 to January 2023: A cross-sectional and longitudinal study design
Source: PLoS One. 2024 Jul 2;19(7):e0306131. doi: 10.1371/journal.pone.0306131 (PMC11219000; doi:10.1371/journal.pone.0306131)
Supplement: Attachment — Submitted filename: Response to Reviewers2.docx [file pone.0306131.s007.docx]

**Table 1:** Responses to Reviewer 1

| **#** | **DESCRIPTION** | **DETAILS ON HOW COMMENT WAS ADDRESSED** | **MANUSCRIPT LINE NUMBER** |
| --- | --- | --- | --- |
|  | **GENERAL** |  |  |
| 1. | The manuscript needs to undergo grammatical revision by a qualified professional. Additionally, the manuscript is not formatted according to the citation and reference style required by the journal, which adopts the "Vancouver Style." Therefore, citations should be numerical and superscripted after the period at the end of each paragraph. | The manuscript has been professionally proofread and the referencing style updated to numerical and superscripted “Vancouver style” citation placed after the period at the end of sentence. | Throughout the document |
| 2. | The article Clinical Characteristics and Factors Associated with Long COVID in Zambia, August 2020 to January 2023: A Mixed Methods Design, presents a very relevant topic in contemporary times. Thank you for the opportunity to review the article and learn from the authors. I make some suggestions so that the article is ready for publication.  I suggest that the study population be addressed in the title and objectives (adults, children, elderly people)? | Thank you for pointing this out. We agree with this comment and have, therefore, revised the title to now read as follows: “Clinical Characteristics and Factors Associated with Long COVID among Post-acute COVID-19 Clinic Patients in Zambia, August 2020 to January 2023: A Cross-sectional and Longitudinal Study Design” | Line 1-2 |
|  | **ABSTRACT** |  |  |
| 3. | In the abstract, in the methods section, it is not clear that it is a mixed methods study. What did the authors consider mixed methods? I suggest not using this “mixed methods” term, as it refers to the use of quantitative and qualitative analyses, considering that two types of quantitative analyzes were adopted in the study (cross-sectional and longitudinal). I suggest removing this term throughout the text and also in the title. | The abstract has been re-written to address concerns raised. The term mixed effects has also been removed | Line 21-51 |
|  | The objective of the summary must be the same as that of the introduction. Review page 12, lines 22 to 25. Review page 14, lines 94 to 96. | Thank you pointing this out. This has been addressed. |  |
|  | **INTRODUCTION** |  |  |
|  | In the introduction I suggest that you address what the study contributes and how it advances in relation to the others. | Thank you for this suggestion. We have since provided information on what the study contributes and how it advances in relation to other in the last paragraph of the introduction section. | Line 91-101 |
|  | **METHODS** |  |  |
|  | On page 15, line 102, Study participants, how old were the study participants? This is important information, as it shows the population studied. | We’ve included information on the age of study participants and indicated it as “all ages”. | Line 110 |
|  | On page 15, on line 119, in Study design, as previously stated, I suggest that the term mixed methods should not be used, considering that both analyzes used were quantitative. I suggest modifying it so that it is a cross-sectional and longitudinal study... | Thank you for pointing this out. We agree with this comment and have, therefore, revised the study to a cross-sectional and longitudinal study design. The title has also been revised accordingly. | Line 129 |
|  | On page 15, in Study design, on lines 119 to 129, the variables are described, I suggest that they be removed to the Study variables and definitions item, with the appropriate explanations of when they were used. | We agree with this suggestion. We’ve since moved the paragraph to the study variables section and approximately explained when they were used. | Line 134-159 |
|  | On page 15, on line 130, in the Study variables and definitions item, it must be made clear to the reader what the outcomes were and what the explanatory variables were. How were the variables categorized? I suggest adding. | Thank you for this suggestion. We have more clearly described the outcome and explanatory variables. Categorization for variables has also been included. | Line 134-159 |
|  | On page 16, on line 153, as described, it is not very clear how to use Pearson's chi-square test considering the asymmetry of the sample. | Thank you for pointing this out. We have now more clearly delineated this statement as relating to the cross-sectional analysis. For the longitudinal analysis, measures of associations tests have been removed in supplementary table S3 due to asymmetry of the sample; the property which is inherent for longitudinal studies. | Line 170; S3 Table |
|  | On page 16, lines 151 to 156, which states The Pearson Chi-square and Kruskal Wallis tests for proportions and Wilcoxon rank sum test for medians were used to estimate the direction and magnitude of association, I suggest reviewing the use of these tests to estimate direction and magnitude of the association, the association measures of the regression models used allow this, but the use of these tests does not, what allows us to analyze are the presence of differences between strata. However, was a post-test used for the square to locate where the difference is? | Thank you for this important comment. We have rephrased this sentence to correctly read as “the Pearson Chi-square and Kruskal Wallis tests for proportions and Wilcoxon rank sum test for medians were used to determine the association of explanatory variables to the outcome”. Statistically significant association were considered at p<0.05. No post-tests were subsequently conducted. | Line 172-174 |
|  | On page 16, in lines 157 to 164, it was not clear whether multivariate analysis considered adjustment for all variables, or whether the authors used adjusted analysis for only some variables. I believe it was a multivariate using the p<0.2 criterion for entering variables into the model, but the way it is described is confusing. Need to review and adjust. IN the multivariate analysis in the final model, what was considered statistically significant (p<0.05)? Make that clear. | We have provided a paragraph under bias to explain how missingness in key variables such as vaccinations status and referral to specialist services had listwise deletion >50% when included at multivariable analysis. These variables were thus not controlled for at multivariable analysis. This is mentioned as a limitation in our study. | Line 192-198 |
|  | On page 16, in lines 157 to 177, the association measures extracted by the models used are not included, both for the cross-sectional and longitudinal analyses, this information is only found in the results. | Thank you for pointing this out. We have rephrased the headings for Table 1 to delineate it more clearly as a descriptive table for the cross-sectional analysis. Similarly, supplementary table S3 is now more clearly labelled longitudinal analysis. Furthermore, the statistical analysis section has be rewritten to provide more clarity. | Line 170-183 |
|  | **RESULTS** |  |  |
|  | I suggest that the titles of tables and figures include the population studied, were the patients children, adults, elderly people? ?Include. | Thank you for this suggestion. As can be seen in Table 1, the study participants were of all age groups (from <29 years up to 60+ years). The study was not specific to population age group. We have, however, rephrased our titles in tables and figure to make it clear who the study population where. | Throughout the document |
|  | In the legend of table 1, some information contained in the legend needs to be included in the methods, such as the significance adopted to analyze the associations using the tests adopted. The question of the denominator and what was calculated. | Thank you for this suggestion. All information, including significant level adopted and definition of variables have been included in the methods section. | Line 135-159; Line179-181 |
|  | On page 20, on lines 223 to 221, where are these results presented? Signal. | Thank you for pointing this out. We have since included Fig 3 as the source of this information. | Line 248-250 |
|  | On page 21, on lines 222 to 228, where are these results presented? Signal. | Thank you for pointing this out. We have since included Fig 3 as the source of this information. | Line 248-250 |
|  | On page 21, on lines 229 to 236, where are these results presented? Signal. | Thank you for pointing this out. We have now referenced Table 1 and S3 Table were this information is drawn from. | Line 253 |
|  | On page 21, on lines 237 to 243, where are these results presented? Signal. | Thank for pointing this out. We have since reference Table 2 and further indicated that is in reference to the cross-sectional analysis. | Line 261 |
|  | This information from table 2 must be included in the methods: and Vaccination status and referral to specialist services were not adjusted for at multivariable analysis due to >10% missingness which resulted in 50.5% listwise deletion. | Thank for this suggestion. This information has been included under bias in the methods section | Line 193-198 |
|  | Throughout the result, I suggest reviewing the use of the term probability, since the Odds Ratio were adopted to arrive at the results. Modify to chance. | Thank you for this suggestion. This has been corrected accordingly | Line 261- |
|  | **DISCUSSION** |  |  |
|  | Throughout the discussion I suggest reviewing the use of the term probability, since the Odds Ratio were adopted to arrive at the results. Modify to chance. | Thank you for this suggestion. We have adopted the use of the chance throughout the discussion and conclusion section. | Line 324-409 |
|  | Organize the order of the discussion according to the results. |  |  |
|  | On page 27, lines 347 to 364, the limitations inherent to cross-sectional and longitudinal studies are not mentioned. Furthermore, it was not clear how representative the sample was for Zambia, as well as the possibility of generalizing these data. These limitations and strengths of this study need to be explored in greater detail, providing a counterpoint. | Thank you for providing this suggestion. We have since added a statement on the representativeness of the study in the discussion section | Line 391-397 |
|  | Regarding the vaccination aspect, the explanations of the vaccines used, depending on their brand, are not clear in the discussion. What is the purpose of this information? | Per initial review process, we were asked to provide more details on vaccination since our study found a non-significant association. This paragraph serves to provide context of the vaccine type received and the scale up in vaccination which could explain the observed effects. |  |
|  | **CONCLUSSION** |  |  |
|  | On page 28, line 362, the conclusion does not respond to the objective of the study. Need to review. | The conclusion has been rewritten to align with the study objectives and study findings | Line 399-409 |

**Table 2: Responses to Reviewer 2**

| **#** | **DESCRIPTION** | **DETAILS ON HOW COMMENT WAS ADDRESSED** | **MANUSCRIPT LINE NUMBER** |
| --- | --- | --- | --- |
|  | **GENERAL** |  |  |
| 1. | Background: does not address the relevance of the study and the knowledge gap. | The background has been re-written to provide a knowledge gap and strengthen the manuscript introduction | Line 94-104 |
| 2. | Methods: it is superficial, important information such as study population, period, study variables and which analyzes were carried out were not included. | Thank you for pointing this out. We have since more clearly delineated the study outcome and explanatory variables | Line 136-161 |
|  | Conclusion: it is not responding to the objective of the study; it must be strictly aligned with the objective. | Thank you for pointing this out. We have since revised the conclusion | Line 399-409 |
|  | **INTRODUCTION** |  |  |
|  | In general, I suggest bringing global epidemiological data on long Covid, and then addressing the context of Africa and Zambia. The authors also mention “Zambia’s experience on the COVID-19 pandemic, however, is characterized by high numbers of SAR-CoV-2 infections despite relatively low numbers of confirmed COVID-19 cases”. But they don't explain why cases weren't confirmed, was it due to a lack of testing? The information is vague and is important to complete.  The authors further state: “While dozens of seroprevalence studies in Africa document the extent of spread of SARS-CoV-2...”. However, they only cite two studies, review. Once again, I emphasize that the lack of studies should not be the justification for carrying out the research, as even if there were few, it may have been sufficient. To review. | The background has been re-written to provide a knowledge gap and strengthen the manuscript introduction | Line 94-104 |
|  | **METHODS** |  |  |
|  | It is necessary to make the methods clearer and more complete, hence the importance of following the checklist for observational studies (STROBE), which must contain the following information: Study design; Setting; Participants; Variables; Data sources/measurement; Bias; Study size; Quantitative variables; Statistical methods. Available at: https://www.strobe-statement.org/checklists/ | Thank you for suggesting the STROBE approach. We have since adopted this approach and revised the manuscript according. | Line 106-210 |
|  | Review the methods and bring all Strobe items in the same sequence. Furthermore, the statistical analysis part does not have a coherent sequence of information. The authors still talk about “regression models” in the plural, but only one model was made. It is not clear.  Another issue that is not clear was the location of the study, was the data collected in all the hospitals mentioned? Review and rewrite. | Thank you for pointing this. On line 126-129, we have provided a statement with regards to how data was routinely abstracted into an electronic databased that was accessed for this data. Data was collected from all the 13 clinics as described in the study setting. | Line 107-111; Line 126-129 |
|  | Did the authors not perform tests for table 2? Was only the 95%CI calculated? Chi-square with Bonferroni correction was not performed? | Thank you for pointing this out. I believe the table being reference is Table 1 since Table is the regression output for the cross-sectional analysis. For the cross-sectional analysis, not pairwise comparisons were made to warrant a Bonferroni correction. For S3 Table however, Bonferroni correction were made | S3 table |
|  | **RESULTS** |  |  |
|  | The following information is not necessary: “...with data entered in the electronic database were included in the analyses”. Because this is methods and not results.  The authors state: “Of these inpatients, 686 (74.6%) were classified as having had severe illness.” What would severe illness be??? It is not clear.  Do the authors have data on how many vaccine doses patients received? If so, I think it's worth adding.  Question: sex, vaccination and referral to a specialized service were not included in the final model? | Thank you for pointing this out. We have since removed the quoted text and revised the sentence to read as follows: “Out of a total 1,359 PAC-19 clinic patients in the cross-sectional study (Fig 1), 548 (40.3%) patients with ≥2 PAC-19 clinic visits were included in the longitudinal sub-analysis.” | Line 212 |
|  | Regarding the authors' statement, "Of these inpatients, 686 (74.6%) were classified as having had severe illness," it is crucial to define what constitutes severe illness for better understanding. | Thank you for pointing this out. We have rephrased this to correctly read as severe acute COVID-19 | Line 42 and |
|  | If available, consider including data on the number of vaccine doses received by patients, as this information could be relevant to the study. | Thank you for this suggestion. A paragraph on number of vaccine doses received by patients have been included. | Line 254-262 |
|  | It is not clear whether sex, vaccination status, and referral to a specialized service were included in the final model. The authors should specify whether these factors were part of the final model in the results section. | Thank you once more for pointing out this. Under bias, we have included statements explaining the reasons the two variables vaccination status and referral to specialized service were not part of the final model. Sex was controlled in the updated model. | Line 194-200 |
|  | **DISCUSSION** |  |  |
|  | The first paragraph in the discussion should serve as a concise summary of the main results, aligning with the study's objective, which will be elaborated on subsequently. Ensure that the discussion follows the sequence of the results. | We are thankful to this suggestion. This paragraph has been re-written to align with the suggestion. | Line 325-332 |
|  | For the fourth paragraph, the mention of bias should be removed. The final paragraph of the discussion is the appropriate space to discuss the study's limitations, including any biases. | Thank you for pointing this out. We have rephased the sentence and removed the word bias. | 385-392 |
|  | Regarding the vaccination aspect, expand the discussion by exploring what other studies reveal about the vaccine. Compare and contrast findings with existing literature. Additionally, delve into the question of how the number of vaccine doses might be associated, highlighting the importance of including and discussing these results. Refer to relevant literature on this topic. | We welcome this suggestion. We have since added text in this regard. | Line 377-379 |
|  | The authors omit a crucial point regarding the local representativeness of the data. It's imperative to acknowledge that the findings may not be extrapolated to other locations or even the entire country, given that the study is based solely on a sample from one institution. Additionally, there is ambiguity regarding the specific hospitals from which the data were collected, requiring clarification for transparency. Please clarify what are the strengths of this work. | Thank you highlighting this omission. The suggested text has been added as suggestd. | Line 389-  392 |
|  | **CONCLUSSION** |  |  |
|  | On page 28, line 362, the conclusion does not respond to the objective of the study. Need to review. | Conclusion has been revised |  |
